# Chitosan–Silk Fibroin Hydrogel Scaffold Incorporating Bioactive *Aloe vera* and Mimosa Complex for Cartilage-Supportive Applications

**DOI:** 10.3390/polym18111406

**Published:** 2026-06-05

**Authors:** Witwisitpong Maneechan, Areeya Tuanchai, Sukunya Ross, Gareth M. Ross, Chatnarong Putthong, Jatuporn Ngoenkam, Yuriko Higuchi, Pensri Charoensit, Jarupa Viyoch

**Affiliations:** 1 Department of Pharmaceutical Technology, Faculty of Pharmaceutical Sciences, Naresuan University, Phitsanulok 65000, Thailand; witwisitpong.ma@nu.ac.th; 2 Center of Excellence in Biomaterials, Department of Chemistry, Faculty of Science, Naresuan University, Phitsanulok 65000, Thailand; areeya.tu@nu.ac.th (A.T.); sukunyaj@nu.ac.th (S.R.); gareth@nu.ac.th (G.M.R.); 3 Division of Applied Thai Traditional Medicine, Faculty of Public Health, Naresuan University, Phitsanulok 65000, Thailand; chatnarongp@nu.ac.th; 4 Department of Microbiology and Parasitology, Faculty of Medical Science, Naresuan University, Phitsanulok 65000, Thailand; jatupornn@nu.ac.th; 5 Department of Drug Delivery Research, Graduate School of Pharmaceutical Sciences, Kyoto University, Kyoto 606-8501, Japan; higuchi.yuriko.6v@kyoto-u.ac.jp; 6 Center of Excellence for Innovation in Chemistry (PERCH-CIC), Faculty of Pharmaceutical Sciences, Naresuan University, Phitsanulok 65000, Thailand

**Keywords:** hydrogel scaffold, chitosan, silk fibroin, aloe vera, mimosa complex, cartilage

## Abstract

A composite hydrogel scaffold comprising chitosan, silk fibroin, *Aloe vera* extract, and Mimosa complex was fabricated and thoroughly characterized. Upon freeze-drying, the scaffolds displayed a uniform cylindrical geometry with a highly porous, interconnected polymeric network. Quantitative image analysis revealed a mean pore diameter of 43.09 ± 2.27 µm alongside an overall porosity of 61.4 ± 6.2%. ATR-FTIR and XRD analyses confirmed successful inclusion of the complex formation and the incorporation of all constituents into the final formulation. The scaffold exhibited a compressive modulus of 46.63 ± 22.71 kPa (dry) and 5.40 ± 3.73 kPa (hydrated), with a swelling ratio of 756.62 ± 114.08%, supporting its suitability for physiological applications. TGF-β3 loading via adsorption yielded an entrapment efficiency of approximately 79.18%, reflecting effective physical immobilization throughout the polymer matrix. Cytocompatibility was subsequently assessed using an indirect contact model combined with an MTT assay, both of which confirmed that TGF-β3-loaded scaffolds exerted no cytotoxic effects on chondrocytes. After 28 days in culture, scanning electron microscopy revealed pronounced cell adhesion, preservation of rounded cell morphology, and ECM deposition along pore walls and throughout interconnected channels. Immunofluorescence analysis further demonstrated a time-dependent accumulation of aggrecan and collagen type II within the three-dimensional scaffold architecture. Collectively, these findings suggest that the developed composite hydrogel scaffold is well-suited for cartilage-related in vitro culture applications.

## 1. Introduction

Osteoarthritis (OA) is a chronic degenerative joint disorder. It is primarily characterized by progressive deterioration of articular cartilage. Key contributing factors include joint injury, altered biomechanical loading, and loss of chondrocyte number and function through apoptosis and senescence [1]. These pathological processes disrupt cartilage homeostasis and ultimately lead to irreversible structural damage, persistent pain, and loss of joint mobility. Articular cartilage is an avascular tissue devoid of nerves and lymphatic vessels. These structural features result in limited nutrient diffusion, poor waste removal, and an extremely low intrinsic capacity for self-repair [2,3]. In early OA, biochemical alterations within the ECM, particularly the degradation of collagen type II and aggrecan, occur prior to visible morphological changes, including cartilage thinning, surface fibrillation, and loss of mechanical integrity [4]. Current clinical treatments primarily address symptoms rather than underlying tissue damage. They fail to restore the structural and functional integrity of articular cartilage. These limitations have driven growing interest in cartilage tissue engineering as a regenerative strategy [5].

Scaffolds are a critical component of cartilage tissue engineering. They must support chondrocyte adhesion, proliferation, and phenotypic maintenance. They must also serve as delivery platforms for bioactive molecules that regulate cartilage regeneration [6]. Hydrogel-based scaffolds have attracted considerable attention due to their high water content and three-dimensional architecture, which closely mimic the native cartilage ECM microenvironment [7]. Among natural polymers, chitosan and silk fibroin are particularly promising candidates. Chitosan is derived from crustacean shells and exhibits excellent biocompatibility, biodegradability, and structural similarity to glycosaminoglycans [8]. However, its poor mechanical strength and rapid degradation restrict its standalone application [9]. Silk fibroin, extracted from *Bombyx mori* cocoons, offers superior mechanical robustness and tunable degradation behavior. However, it lacks sufficient intrinsic bioactivity to promote chondrogenic responses [10]. When combined, these polymers generate synergistic effects. They improve mechanical stability, porosity, and chondrogenic ECM production, including collagen type II and aggrecan, compared to single-polymer systems [11].

Plant-derived bioactive compounds have been increasingly incorporated into biomaterial scaffolds to enhance biological functionality and regenerative performance [12,13]. *Aloe vera* gel extract is rich in glucomannan and acemannan. These constituents provide anti-inflammatory, antioxidant, and ECM-mimetic properties. They promote cell migration, proliferation, and ECM production while suppressing inflammatory responses [14]. When combined with natural polymeric matrices, *Aloe vera* extracts enhance cellular proliferation and early ECM formation in vitro, supporting a regenerative microenvironment [15]. In parallel, preventing microbial contamination remains essential for scaffold safety and clinical translation. Orientin-containing extracts from *Mimosa* species are rich in flavonoids and polyphenolic constituents. These compounds exhibit broad-spectrum antibacterial and antioxidant activities [16,17]. Beyond antimicrobial effects, orientin demonstrates chondroprotective potential by suppressing inflammatory signaling pathways, including PI3K/AKT/NF-κB and MAPK cascades. These effects reduce cartilage degradation in cellular and animal models of OA [18,19,20]. The integration of these plant-derived bioactives into chitosan/silk fibroin hydrogels therefore represents a rational strategy to simultaneously enhance microbial safety and support cartilage-specific cellular function.

Transforming growth factor-beta 3 (TGF-β3) is among the most widely studied chondrogenic growth factors. TGF-β3 promotes chondrocyte redifferentiation and stimulates production of collagen type II and aggrecan within hydrogel scaffolds [21,22]. Its controlled delivery provides sustained chondrogenic stimulation, which is particularly important in avascular cartilage tissue where systemic delivery is ineffective.

In this study, we developed a biodegradable chitosan/silk fibroin hydrogel scaffold functionalized with *Aloe vera* gel extract and orientin-containing Mimosa extract, and loaded with TGF-β3, for cartilage tissue engineering applications. The hydrogel was designed to form a porous and interconnected three-dimensional network under physiological conditions. This architecture supports chondrocyte adhesion, viability, and phenotypic maintenance. We hypothesized that the synergistic integration of these natural polymers and bioactive components would enhance chondrocyte viability and cartilage-specific ECM production, ultimately promoting functional cartilage regeneration.

## 2. Materials and Methods

### 2.1. Materials and Reagents

Chitosan derived from shrimp shells (molecular weight range 10^5^–10^6^ Da; degree of deacetylation exceeding 90%) was purchased from Sinudom Agriculture Products Ltd. (Suratthani, Thailand). Yellow silkworm cocoons of *Bombyx mori* (Nang Laai strain) were kindly provided by the Center for Excellence in Silk, Nakhon Pathom Province, Thailand. Fresh *Aloe vera* and *Mimosa pudica* plant materials were harvested from local cultivation sites in Phitsanulok and Chiang Mai Provinces, Thailand, respectively.

Regarding chemical reagents, sodium bicarbonate, phosphate-buffered saline (PBS), and 88% lactic acid solution were procured from Sigma-Aldrich Chemie GmbH (Steinheim, Germany). Di-sodium hydrogen phosphate (Na_2_HPO_4_) was obtained from Elago Enterprises Pty Ltd. Calcium chloride (CaCl_2_), sodium hydroxide (NaOH), and ammonium sulfate ((NH_4_)_2_SO_4_) were supplied by RCI Labscan (Bangkok, Thailand). A dialysis membrane with a standard RC tubing format (MWCO: 6–8 kDa) was sourced from Spectrum Laboratories, Inc. (Rancho Dominguez, CA, USA).

Additional reagents and consumables included Pierce™ BCA Protein Assay Kit (Thermo Fisher Scientific Inc., Rockford, IL, USA), Dulbecco’s Modified Eagle’s Medium (DMEM), fetal bovine serum (FBS), and 0.25% trypsin/0.01 M EDTA solution (Sigma-Aldrich Co., St. Louis, MO, USA). Gibco™ Penicillin–Streptomycin solution (10,000 U/mL), Thiazolyl Blue Tetrazolium Bromide (MTT) as a cell viability reagent, and paraformaldehyde were obtained from Thermo Fisher Scientific Inc. and Sigma-Aldrich, respectively. ProLong™ Diamond Antifade Mountant with DAPI was also acquired from Thermo Fisher Scientific Inc.

### 2.2. Preparation of Natural Polymer and Plant-Derived Extracts

#### 2.2.1. Silk Fibroin and Aloe Vera Gel Extraction

Silk fibroin extraction from *Bombyx mori* (Nang-Lai strain) cocoons was performed based on a modified version of the procedure reported by Phimnuan et al. [23]. Briefly, cocoons were degummed by boiling in 25 mM Na_2_CO_3_ solution for 30 min to remove sericin, followed by thorough rinsing with deionized water and air-drying. The degummed silk fibers were subsequently dissolved in 6 M CaCl_2_ solution at 90 °C for 90 min. The resulting solution was filtered to remove undissolved residues and dialyzed against deionized water using a dialysis membrane with a molecular weight cut-off (MWCO) of 6–8 kDa for 72 h, with water changes every 12 h, to remove residual calcium ions and salts. The purified silk fibroin solution was subsequently lyophilized to obtain silk fibroin powder.

*Aloe vera* gel extract was prepared following the same modified protocol [23]. The inner gel was collected from fresh *Aloe vera* leaves, homogenized to obtain a uniform gel suspension, and bioactive components were precipitated by the addition of ammonium sulfate ((NH_4_)_2_SO_4_). The resulting precipitate was recovered by centrifugation and dialyzed against deionized water using the same membrane (MWCO 6–8 kDa) for 72 h with water changes every 12 h to remove residual salts. The purified extract was lyophilized to obtain *Aloe vera* gel extract powder. All extracted materials were stored in a desiccator at room temperature until further use.

The physicochemical characterization of both extracts, including protein content, molecular weight distribution, and chemical composition, has been previously reported [24]. As identical extraction protocols and preparation conditions were employed in the present study, these analyses were not repeated, and the extracts were assumed to exhibit comparable physicochemical characteristics.

#### 2.2.2. *Mimosa pudica* Extraction

*Mimosa pudica* stems and leaves were washed, air-dried, and ground into a fine powder. The powdered material was extracted by maceration in 95% ethanol at a solid-to-solvent ratio of 1:10 (*w*/*v*) for 72 h at room temperature. The extract was filtered, and the solvent was removed under reduced pressure to obtain the crude extract. To ensure quality and consistency across batches, the content of orientin (a major active constituent of *Mimosa pudica*) was quantified by High-Performance Liquid Chromatography (HPLC). An Agilent 1260 Infinity series system equipped with a ZORBAX SB-C18 analytical column (250 × 4.6 mm, 5 μm particle size) was used for analysis. The mobile phase consisted of 0.1% orthophosphoric acid (mobile phase A) and acetonitrile (mobile phase B) delivered in a gradient system as follows: 0 min (90:10), 5 min (80:20), 10 min (73:27), 15 min (65:35), 20 min (55:45), 30 min (20:80), and 35 min (90:10, *v*/*v*). The flow rate was 0.8 mL/min, injection volume was 20 μL, and column temperature was maintained at 35 °C. Detection was performed using a diode array detector (DAD) at wavelengths of 254, 280, 320, 340, and 370 nm. The total run time was 35 min with a post-run time of 10 min. This analysis confirmed that each batch contained a comparable level of active compounds prior to further use.

To improve solubility and stability, the crude extract was complexed with hydroxypropyl-β-cyclodextrin (HPβCD) according to a previously reported method [25]. Briefly, the crude extract was mixed with HPβCD at a weight ratio of 1:5 (crude extract:HPβCD) using ethanol as a co-solvent and ground until a homogeneous paste was obtained. The complex was dried in a desiccator, sieved to obtain particles ≤ 200 μm, and stored in the dark at room temperature until use.

### 2.3. Fabrication of CS/SF/AV/Mimosa Complex Hydrogel Scaffolds

#### 2.3.1. Scaffold Fabrication

A hydrogel scaffold containing CS, SF, AV, and Mimosa complex was prepared as a three-dimensional platform for biological evaluation. Each component was individually prepared prior to combining into the final formulation.

Chitosan (degree of deacetylation 90–95%, molecular weight 10^5^–10^6^ Da) was dissolved in 0.5 M lactic acid at approximately 4.3% (*w*/*v*) under magnetic stirring until fully dissolved. The pH was then adjusted to 5–6 using 10% (*w*/*v*) sodium bicarbonate solution. Separately, SF (2.7% *w*/*v*, protein content 88–98%) and AV extract (0.075% *w*/*v*, protein content 4–8%) were co-dissolved in deionized water (type II, 15 MΩ·cm) at a weight ratio of 36:1 until a homogeneous solution was obtained. The Mimosa complex prepared in Section 2.2.2 was dissolved in deionized water to prepare a stock solution at 100 mg/mL. The solution was filtered through a 0.22 µm membrane filter and stored at 4 °C until use.

To prepare the hydrogel, the SF/AV solution was mixed with the CS solution at a weight ratio of 40:36:1 (CS:SF:AV) under gentle stirring. The Mimosa complex stock solution (1000 µL) was then added to reach a final concentration of 5 mg/mL. Based on the HPLC-determined orientin content of the crude extract (2.75 ± 0.02 mg/g extract) and the complexation ratio of 1:5 (crude extract:HPβCD), the estimated orientin content in the final hydrogel formulation was approximately 2.3 µg/mL. Na_2_HPO_4_ was added as a crosslinking agent at 38–40 wt% relative to total polymer content and stirred until a uniform precursor was formed. The precursor was frozen at −80 °C and freeze-dried to obtain porous scaffolds, which were kept at 4–8 °C prior to use.

#### 2.3.2. Pore Size and Porosity Analysis

The pore size and porosity of the hydrogel scaffolds were analyzed using ImageJ software version 1.54g (National Institutes of Health, Bethesda, MD, USA) based on FESEM images of scaffold cross-sections obtained at a magnification of ×200. Scaffold cross-sections were examined using field emission scanning electron microscopy (FESEM; Thermoscientific Apreo S, Waltham, MA, USA) at an accelerating voltage of 5.00 kV, working distance of 9.1 mm, spot size of 7.0, and beam current of 50 pA under high-vacuum conditions using a secondary electron (ETD) detector. Samples were sputter-coated with gold prior to imaging. Three images per sample (*n* = 3) were used for analysis, with a minimum of 100 pores measured per group. The images were calibrated using the scale bar and converted to grayscale prior to thresholding to distinguish pore regions from the scaffold matrix. The thresholded images were binarized and analyzed using the Analyze Particles function in ImageJ. The equivalent pore diameter was calculated for each pore assuming a circular geometry, while pores located at image boundaries or associated with artifacts were excluded from the analysis. Porosity was calculated as the percentage of pore area relative to the total image area, determined from the binary FESEM images. The pore size distribution was presented as a histogram with a fitted Gaussian distribution curve.

#### 2.3.3. Chemical Analysis

Functional groups present in individual materials and composite hydrogel scaffolds were identified by Attenuated Total Reflectance-Fourier Transform Infrared (ATR-FTIR) spectroscopy using a Frontier spectrometer (PerkinElmer, Waltham, MA, USA). Spectra were recorded over a wavenumber range of 4000–400 cm^−1^ at a resolution of 2 cm^−1^, with 64 scans per sample. The crystallinity of each sample was examined by X-ray diffraction (XRD) using a D2 PHASER diffractometer (Bruker, Billerica, MA, USA). Diffraction patterns were obtained with Cu Kα radiation (λ = 1.5406 Å) over a 2θ range of 5–50°.

#### 2.3.4. Compressive Mechanical Testing

Compressive mechanical properties were evaluated under both dry and hydrated conditions following a previously described protocol [24]. Briefly, compression testing was performed using a TA.XT plus Texture Analyser (Microsystems Ltd., London, UK) at a rate of 1 mm/min with a 30 kg load cell. Specimens were compressed to 60% of their original height. Compressive modulus was calculated from the initial linear region of the stress–strain curve (0–2% strain), and compressive strength was recorded at 20% deformation. For hydrated testing, specimens were immersed in PBS for 24 h prior to analysis. All measurements were performed in triplicate (*n* = 3).

#### 2.3.5. Swelling Study

Swelling behaviour was assessed according to Felfel et al. [26]. Briefly, dried scaffolds were weighed (W_0_) and immersed in PBS (pH 7.4) at 37 °C for 24 h. Specimens were then removed, blotted with filter paper to remove surface moisture, and reweighed (W_1_). The swelling ratio was calculated as ((W_1_ − W_0_)/W_0_) × 100. All measurements were performed in triplicate (*n* = 3).

### 2.4. Growth Factor Loading by TGF-β3 Adsorption

#### 2.4.1. Preparation of TGF-β3 Solution

Recombinant human transforming growth factor-β3 (TGF-β3) was reconstituted in phosphate-buffered saline (PBS) containing 0.1% (*w*/*v*) bovine serum albumin (BSA) to minimize non-specific adsorption. A stock solution was prepared according to the manufacturer’s instructions and diluted with PBS/0.1% BSA to obtain a working concentration of 15 ng/mL. This concentration was selected to provide sufficient protein availability for adsorption onto the hydrogel scaffolds while allowing for the accurate quantification of unadsorbed TGF-β3 within the linear detection range of the ELISA assay.

#### 2.4.2. Adsorption of TGF-β3 onto Hydrogel Scaffolds

Freeze-dried hydrogel scaffolds (diameter: 14.7 mm; thickness: 3 mm) were sterilized by immersion in 70% ethanol for 15 min, followed by three washes with sterile PBS to remove residual ethanol. The sterilized scaffolds were then loaded with TGF-β3 using an adsorption-based method. Briefly, each scaffold was immersed in 1 mL of TGF-β3 working solution (15 ng/mL) and incubated at 4 °C under gentle agitation for 24 h to allow protein adsorption. After incubation, the scaffolds were removed, and the remaining supernatant was collected for quantification of unadsorbed TGF-β3. Scaffolds incubated in PBS/0.1% BSA without TGF-β3 under identical conditions were used as non-loaded controls. Three scaffolds per group (*n* = 3) were used for all adsorption experiments.

#### 2.4.3. Quantification of Unadsorbed TGF-β3 and Standard Curve Construction

Unadsorbed TGF-β3 remaining in the collected supernatant was measured using a Human TGF-β3 ELISA kit (ab272203, Abcam, Cambridge, UK) following the manufacturer’s instructions. Briefly, 50 µL of each standard or sample was pipetted into antibody-coated microplate wells, followed by the addition of 50 µL antibody cocktail. The plate was then incubated at room temperature for 1 h and washed three times with 350 µL of 1× wash buffer. TMB substrate solution (100 µL) was added to each well and allowed to react for 10 min under dark conditions. The enzymatic reaction was terminated by adding 100 µL of stop solution, and absorbance was read at 450 nm with a microplate reader.

For standard curve construction, recombinant human TGF-β3 standards supplied in the kit were prepared by serial dilution over a concentration range of 0–12 ng/mL. Blank-corrected absorbance values were plotted against the corresponding standard concentrations, and TGF-β3 levels in test samples were calculated by linear regression. All assays were conducted in triplicate (*n* = 3).

#### 2.4.4. Determination of Entrapment Efficiency

The entrapment efficiency (EE) of TGF-β3 was calculated using the following equation: EE (%) = ((Initial amount of TGF-β3-Residual amount in supernatant/Initial amount of TGF-β3) × 100).

### 2.5. Chondrocytes Culture and Seeding

A human primary chondrocyte cell line (HUM-iCell-s018, iCell Bioscience Inc., Shanghai, China) was used in this study. Cells were cultured in the Primary Chondrocyte Culture System (PriMed-iCell-020, iCell) at 37 °C in a humidified atmosphere containing 5% CO_2_. The culture medium was changed every 3–4 days. When cells reached 70–80% confluency, they were detached using Trypsin-EDTA solution (Sigma-Aldrich Co., St. Louis, MO, USA) and subcultured. Cells at passage five and above were seeded onto scaffold constructs in culture plates and maintained under standard culture conditions.

### 2.6. In Vitro Cytocompatibility Assessment (Indirect Contact Method)

Cell viability was evaluated using an indirect contact method with a transwell system and an MTT colorimetric assay. This approach was employed to assess the effects of soluble components and released growth factors from the scaffolds without direct physical contact with the cells.

Prior to the experiment, the scaffolds were sterilized by immersion in 70% (*v*/*v*) ethanol for 15 min, followed by thorough rinsing three times with sterile PBS. The sterilized scaffolds were subsequently preconditioned by incubation in complete culture medium for 12 h at 37 °C to ensure medium saturation. The scaffolds were then loaded with TGF-β3 (15 ng/mL) as described in the growth factor loading section.

Cells were seeded in the lower wells of tissue culture plates at a density of 3 × 10^4^ cells per well and cultured for 24 h to allow cell attachment under standard conditions (37 °C, 5% CO_2_, humidified atmosphere). After cell attachment, the culture medium was replaced with fresh medium, and transwell inserts (6.5 mm diameter, 0.4 μm pore size) containing the TGF-β3-loaded scaffolds were placed into the wells, enabling indirect exposure of the cells to scaffold-derived soluble factors through the shared culture medium.

Cells cultured without scaffolds in transwell inserts served as scaffold-free controls. All cultures were maintained under standard conditions throughout the experiment. Cell viability was assessed after 24 and 72 h of indirect exposure to the TGF-β3-loaded scaffolds.

At each time point, the culture medium was removed and replaced with fresh medium containing 0.5 mg/mL MTT solution. Cells were then incubated at 37 °C for 4 h to allow metabolically active cells to reduce MTT into insoluble formazan crystals. After incubation, the medium was discarded and the formazan crystals were dissolved in dimethyl sulfoxide (DMSO). The absorbance of each well was measured at 540 nm using a microplate reader.

All experiments were performed in triplicate (*n* = 3). Cell viability was normalized to the scaffold-free control at the corresponding time point and expressed as a percentage, with the control defined as 100%.

### 2.7. Biological Evaluation on Hydrogel Scaffold

Cell morphology on the scaffold surface was assessed by field emission scanning electron microscopy (FESEM; Thermoscientific Apreo S). After 28 days of culture, the scaffold was fixed in 4% paraformaldehyde (PFA) in PBS. The fixed samples were then freeze-dried, sputter-coated with a thin gold layer, and examined under field emission scanning electron microscopy (FESEM; Thermoscientific Apreo S) at an accelerating voltage of 5.00 kV, working distance of 9.1–9.4 mm, spot size of 7.0, and beam current of 50 pA under high-vacuum conditions using a secondary electron (ETD) detector. Images were acquired at magnifications of 2000× and 5000×.

Immunofluorescence staining was used to assess cartilage-specific extracellular matrix proteins in chondrocytes grown on hydrogel scaffolds. At day 28, samples were fixed in 4% PFA in PBS (pH 7.4) for 20 min at room temperature and then washed three times with PBS.

Cell membranes were permeabilized with 0.1% Triton X-100 in PBS for 10 min at room temperature, followed by PBS washing. Non-specific binding sites were then blocked with 2% bovine serum albumin (BSA) in PBS for 1 h at room temperature.

Samples were incubated overnight at 4 °C in a humidified chamber with the following primary antibodies diluted in antibody dilution buffer: mouse monoclonal anti-aggrecan antibody (clone BC-3, Invitrogen) and rabbit polyclonal anti-collagen type II antibody (PA5-99159, Thermo Fisher Scientific; 1:150 dilution).

After primary antibody incubation, samples were washed three times with PBS and incubated with species-specific fluorophore-conjugated secondary antibodies for 1–2 h at room temperature in the dark. Aggrecan was detected using Alexa Fluor 488–conjugated anti-mouse IgG, while collagen type II was detected using Alexa Fluor 647–conjugated anti-rabbit IgG (both at 1:500 dilution).

Nuclei were counterstained with DAPI using ProLong™ Diamond Antifade Mountant. Samples were mounted on glass slides and cured for 24 h at room temperature in the dark. Fluorescence images were acquired within 24–48 h using an Axio Observer Z1 inverted fluorescence microscope (Carl Zeiss AG, Oberkochen, Germany) under identical imaging settings for all samples.

### 2.8. Statistical Analysis

All data are reported as mean ± SD. Group differences were evaluated using an unpaired two-tailed Student’s *t*-test. A *p*-value of less than 0.05 was considered statistically significant. All calculations were performed with SPSS software version 19 (IBM Corp., Armonk, NY, USA).

## 3. Results and Discussion

### 3.1. Appearance, Morphology and Pore Characteristics of Hydrogel Scaffolds

**Figure 1:**
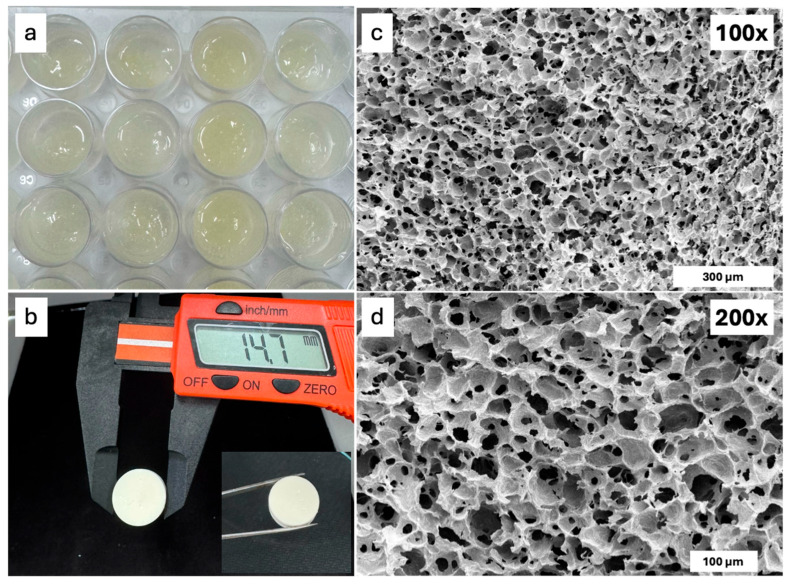
Physical appearance and morphology of the hydrogel and freeze-dried scaffolds. (**a**) Hydrogel precursor in a 24-well plate; (**b**) cylindrical freeze-dried scaffold; (**c**,**d**) FESEM images of the scaffold showing a porous structure at 100× and 200× magnification (scale bars = 300 and 100 µm, respectively).

The appearance and morphology of the hydrogel and freeze-dried scaffolds are shown in Figure 1. The hydrogel exhibited a homogeneous and translucent appearance when cast in a 24-well plate prior to freeze-drying (Figure 1a). After freeze-drying, the scaffolds maintained a well-defined cylindrical shape with a uniform structure (Figure 1b).

FESEM analysis revealed that the freeze-dried scaffolds possessed a highly porous and interconnected microstructure. At low magnification (100×), pores were uniformly distributed throughout the scaffold matrix (Figure 1c), while higher magnification images (200×) demonstrated thin pore walls and an open three-dimensional network (Figure 1d).

**Figure 2:**
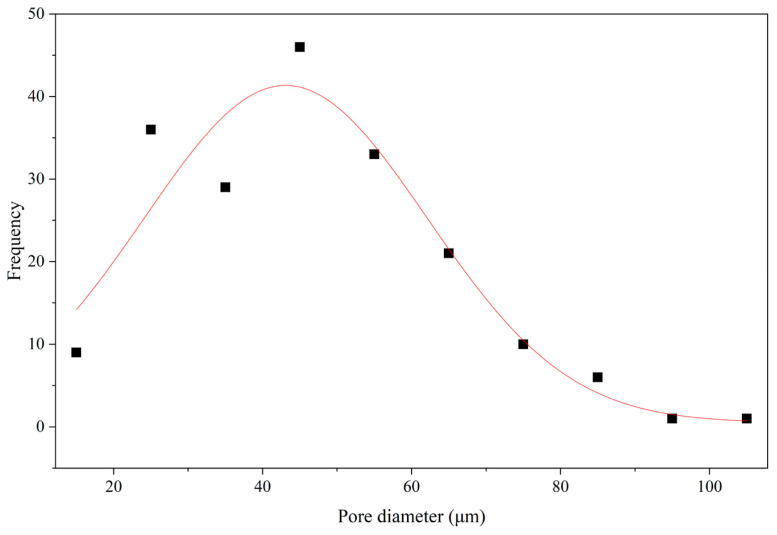
Pore diameter distribution of the hydrogel scaffolds determined from FESEM images. The histogram was generated based on pooled pore size measurements from three independent FESEM images (*n* = 3 images per sample; minimum 100 pores per group) obtained using ImageJ software and fitted with a normal distribution curve. The mean pore diameter derived from Gaussian fitting was 43.09 ± 2.27 μm (mean ± SD). Filled squares (■) represent observed data points, and the red line represents the fitted Gaussian distribution curve.

Quantitative pore size analysis was performed based on FESEM images using ImageJ software. The pore diameter distribution of the hydrogel scaffolds ranged from approximately 15 to 105 µm, as shown in Figure 2. The pore size histogram exhibited a unimodal distribution and was well fitted by a Gaussian function. Nonlinear curve fitting revealed a mean pore diameter (xc) of 43.09 ± 2.27 µm, with a coefficient of determination (R^2^ = 0.903). In addition, the scaffolds exhibited an overall porosity of 61.4 ± 6.2%, as determined from image-based analysis.

The freeze-dried hydrogel scaffolds exhibited a porous and interconnected microarchitecture, which is a typical feature of lyophilized hydrogel systems. This structure is considered beneficial for tissue engineering applications, as it allows cell infiltration and supports the transport of nutrients, oxygen, and metabolic waste throughout the scaffold [27]. The unimodal pore size distribution with a mean pore diameter of approximately 43 µm falls in the lower end of the range reported to support chondrocyte attachment and proliferation in cartilage tissue engineering scaffolds. Lien et al. [28] reported that pore sizes of 50–200 µm were associated with favorable chondrocyte behavior and ECM secretion in gelatin-based scaffolds. Although the mean pore diameter in the present study is slightly below this range, it may still provide a suitable three-dimensional microenvironment for extracellular matrix deposition [29]. Moreover, the relatively high porosity is expected to compensate for the smaller pore diameter by enhancing mass transport and providing sufficient space for matrix accumulation, which is critical for cartilage tissue formation [30]. Collectively, these architectural features indicate that the fabricated hydrogel scaffolds provide a favorable three-dimensional microenvironment for cartilage tissue engineering applications.

### 3.2. FTIR Spectra and XRD Patterns

**Figure 3:**
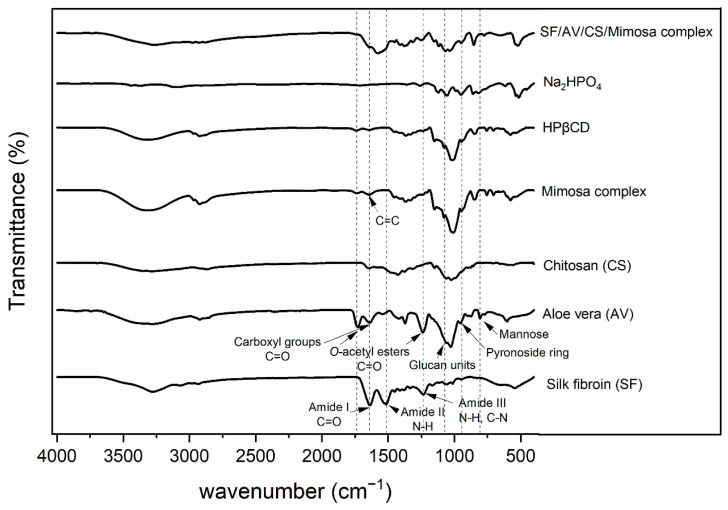
FTIR spectra of pure components and composite scaffold.

The FTIR spectra of pure components and the composite scaffold are presented in Figure 3. The SF spectrum showed characteristic amide bands at approximately 1643 cm^−1^ (Amide I, C=O stretching), 1515 cm^−1^ (Amide II, N-H bending), and 1234 cm^−1^ (Amide III, N-H and C-N stretching). The AV spectrum exhibited polysaccharide-related absorption bands, including carboxyl groups at 1644 cm^−1^, O-acetyl esters at 1730 and 1238 cm^−1^, glucan units at 1058 cm^−1^, mannose residues at 807 cm^−1^, and pyronoside ring vibrations at 955 cm^−1^. The CS spectrum displayed a broad O-H and N-H stretching band in the 3500–3000 cm^−1^ region with glycosidic backbone absorptions in the 1100–1000 cm^−1^ region. The pure HPβCD spectrum showed a broad O-H stretching band at 3322 cm^−1^, C-H stretching at 2968 and 2922 cm^−1^, C-O-C glycosidic stretching at 1150 and 1079 cm^−1^, and C-O stretching at 1021 cm^−1^. The Mimosa complex spectrum exhibited a similar overall profile to pure HPβCD; however, a notably higher intensity of the C=C stretching band at 1644 cm^−1^ was observed, reflecting the aromatic flavonoid skeleton of *Mimosa pudica* constituents encapsulated within the cyclodextrin cavity [31].

The molecular interactions within the composite scaffold were further examined by comparing peak positions between individual components and the final formulation. The broad O-H and N-H stretching band of chitosan in the 3500–3000 cm^−1^ region showed broadening in the composite spectrum, suggesting the formation of intermolecular hydrogen bonding between the hydroxyl and amino groups of chitosan and the carbonyl groups of silk fibroin, consistent with previous reports on CS/SF composite scaffolds [32]. The ionic crosslinking mechanism between chitosan and Na_2_HPO_4_ is reflected by the retention of characteristic phosphate absorption bands in the composite spectrum, where the negatively charged phosphate groups of Na_2_HPO_4_ interact electrostatically with the protonated amino groups (-NH_3_^+^) of chitosan, forming an ionically crosslinked network [33]. This crosslinking mechanism is consistent with the XRD data, which showed residual crystalline peaks attributable to Na_2_HPO_4_ retaining its structure after crosslinking. The spectrum of the composite scaffold (SF/AV/CS/Mimosa complex) retained the characteristic absorption peaks of all individual components without significant alteration, confirming the successful incorporation of all constituents into the final formulation [34].

**Figure 4:**
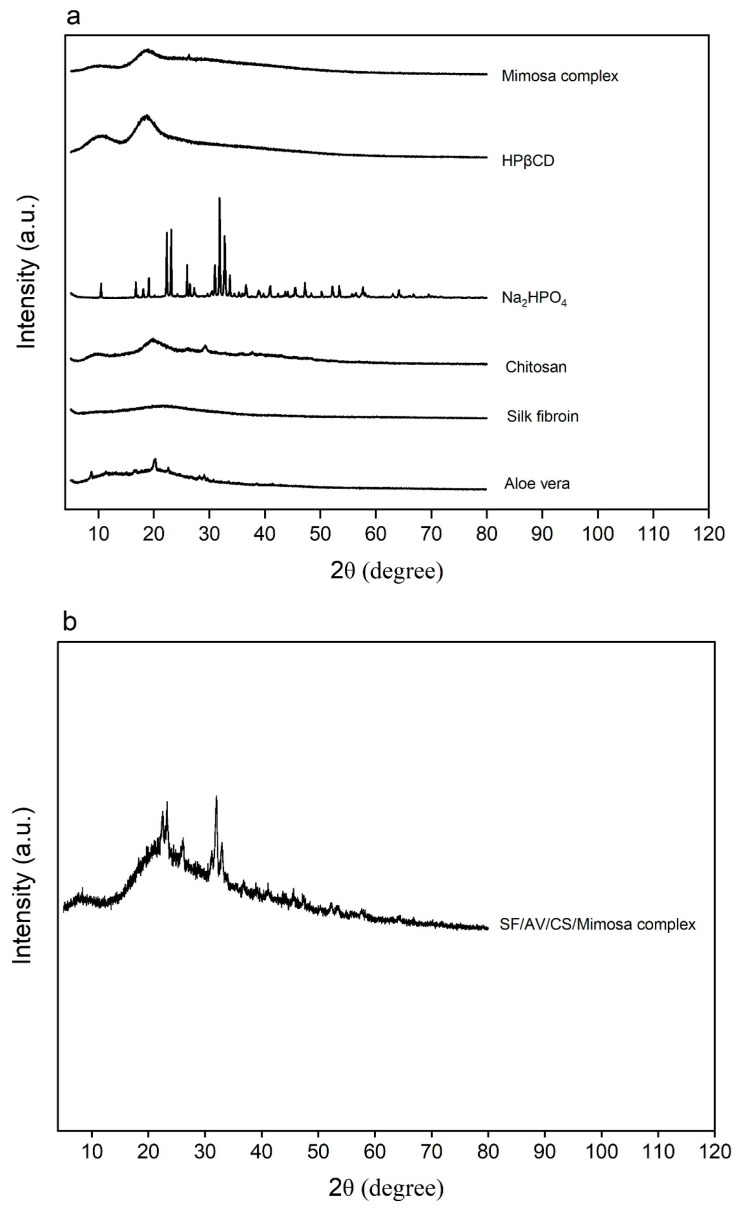
X-ray diffraction (XRD) patterns of individual components and composite formulation. (**a**) XRD patterns of Mimosa–HPβCD inclusion complex, hydroxypropyl-β-cyclodextrin (HPβCD), disodium hydrogen phosphate (Na_2_HPO_4_), chitosan, silk fibroin, and *Aloe vera*. (**b**) XRD pattern of the SF/AV/CS/Mimosa complex composite.

The XRD patterns of individual components and the composite scaffold are presented in Figure 4a,b. SF and AV exhibited broad, diffuse peaks with low intensity, confirming their amorphous nature. CS showed broad peaks with low intensity at approximately 10°, 20°, and 29°, indicating the presence of both crystalline and amorphous regions. HPβCD exhibited a broad diffraction pattern with no distinct crystalline peaks, consistent with its amorphous character. In contrast, Na_2_HPO_4_, which served as a crosslinker in the formulation, displayed several sharp and intense peaks at 2θ angles around 20° and 30°, indicative of a highly crystalline structure. The Mimosa complex exhibited a broad amorphous pattern closely resembling that of pure HPβCD, with the complete absence of sharp crystalline peaks otherwise expected from the phytochemical constituents of *Mimosa pudica*, indicating that the active compounds lost their crystallinity upon inclusion complex formation with HPβCD [35,36]. The XRD pattern of the composite scaffold (Figure 4b) retained the broad amorphous background of the individual biopolymer components, with residual sharp peaks in the 2θ range of 20–30° attributable to the Na_2_HPO_4_ crosslinker retaining its crystalline structure after crosslinking.

The predominantly amorphous nature of the composite scaffold represents a favorable characteristic for cartilage-supportive applications, as amorphous polymer matrices generally exhibit higher water uptake capacity and more favorable bioactive compound release profiles compared to crystalline materials [37]. This semi-crystalline character provides a biomimetic environment more closely resembling the partially ordered structure of the natural extracellular matrix in cartilage tissue, which is known to enhance cellular adhesion and chondrocyte interaction [6]. Furthermore, the absence of new crystalline peaks in the composite XRD pattern confirms that no undesirable crystalline phases were formed during scaffold fabrication, supporting the physicochemical compatibility of all components within the formulation.

### 3.3. Compressive Mechanical Properties

**Figure 5:**
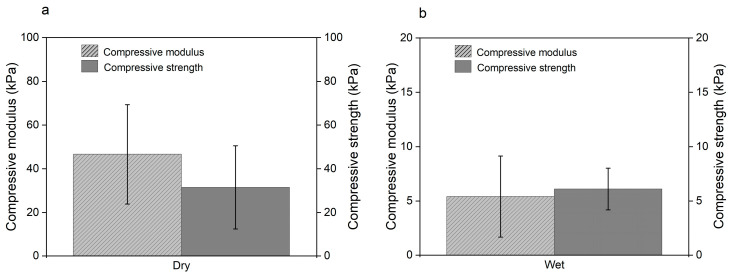
Compressive properties of the scaffold under (**a**) dry and (**b**) hydrated conditions following 24 h immersion in PBS. Data represent mean ± SD (*n* = 3).

The compressive properties of the scaffold were evaluated under both dry and hydrated conditions (Figure 5). Under dry conditions, the scaffold exhibited a compressive modulus of 46.63 ± 22.71 kPa and a compressive strength of 31.48 ± 19.00 kPa. Following 24 h immersion in PBS, the hydrated scaffold showed a compressive modulus of 5.40 ± 3.73 kPa and a compressive strength of 6.09 ± 1.97 kPa. The reduction in compressive modulus upon hydration reflects the characteristic viscoelastic behaviour of hydrogel scaffolds, attributed to water uptake within the polymer matrix. The wet-state compressive modulus (5.40 ± 3.73 kPa) is comparable to values reported for hydrogel scaffolds used in cartilage tissue engineering [7,24], suggesting adequate mechanical stability to support chondrocyte culture under physiological conditions.

### 3.4. Swelling Behavior

The swelling ratio of the CS/SF/AV/Mimosa scaffold was 756.62 ± 114.08% after immersion in PBS (pH 7.4) at 37 °C for 24 h. This high water uptake capacity reflects the hydrophilic nature of the composite components, including chitosan, silk fibroin, and *Aloe vera* extract. The incorporation of the Mimosa complex may further enhance water penetration, as HPβCD possesses a hydrophilic outer surface. This value is higher than that previously reported by Maneechan et al. [24] for CS40/SF/AV scaffolds (approximately 270%), suggesting that the addition of the Mimosa complex introduces additional hydrophilic domains within the scaffold matrix. Adequate swelling capacity supports nutrient diffusion and chondrocyte viability, while the porous architecture generated by freeze-drying facilitates fluid infiltration throughout the construct.

### 3.5. Analysis of Growth Factor Loading by TGF-β3 Adsorption

The standard calibration curve for human transforming growth factor-β3 (TGF-β3), generated using the Human TGF-β3 ELISA kit, exhibited excellent linearity over the concentration range of 0–12 ng/mL, with a regression equation of y = 0.2523x − 0.0138 and a coefficient of determination of R^2^ = 0.9996, confirming the reliability of the assay for quantitative analysis.

Following adsorption onto the hydrogel scaffolds, the concentration of unadsorbed TGF-β3 in the supernatant was quantified by ELISA. Residual TGF-β3 concentrations ranged from 2.51 to 3.86 ng/mL, with a mean value of 3.12 ± 0.51 ng/mL (*n* = 3). Based on an initial TGF-β3 concentration of 15 ng/mL, the entrapment efficiency (EE) of TGF-β3 was calculated to be 79.18%, indicating effective adsorption of the growth factor onto the scaffold matrix.

The efficient adsorption of TGF-β3 onto the polymeric hydrogel scaffold demonstrated in this study highlights the suitability of the adsorption-based loading strategy for growth factor immobilization. Physical interactions between the scaffold matrix and TGF-β3 enable high loading efficiency while avoiding chemical modification, thereby preserving the bioactivity of the growth factor [38,39]. Such adsorption-mediated systems are widely recognized for their potential to support localized and sustained growth factor delivery in tissue engineering applications [40].

Beyond adsorption performance, assessment of cytocompatibility is a critical step in validating the biological safety of growth-factor-loaded polymeric scaffolds prior to further biological application. Soluble components released from the scaffold matrix, including degradation products and released TGF-β3, may exert time-dependent effects on cellular behavior and viability. Therefore, in vitro cytotoxicity testing using standardized methods is necessary to confirm that neither the scaffold matrix nor the adsorption-based loading approach induces adverse cellular effects [41,42].

In this context, an indirect contact cytotoxicity model using a transwell system is particularly appropriate for evaluating cell responses to scaffold-derived soluble factors, as it eliminates confounding effects from direct physical contact between cells and the scaffold. This approach is consistent with the indirect contact test methodology described under ISO 10993-5 [41], which specifically identifies indirect contact testing as suitable for assessing the potential toxicity of leachable substances released from polymeric biomaterials. The use of this standardized indirect contact approach has been applied in the cytotoxicity assessment of various polymeric scaffolds and composite biomaterials [43]. Therefore, in vitro cytotoxicity assessment under indirect contact conditions was conducted to further validate the cytocompatibility of the TGF-β3-loaded scaffolds prior to subsequent biological evaluation.

Although TGF-β3 adsorption efficiency was demonstrated in the present study, quantitative release kinetics data were not determined. Previous studies have reported that growth factors loaded onto hydrogel scaffolds via adsorption typically exhibit an initial burst release, followed by sustained release over an extended period [44]. For instance, TGF-β loaded by non-specific adsorption was released approximately 70% within the first week [45], while adsorption-based delivery systems have demonstrated retention of over 50% of the loaded growth factor within the scaffold after 21 days [46]. Furthermore, porous scaffolds have been shown to support prolonged TGF-β3 release of up to 50% over 42 days, which was sufficient to promote chondrogenic marker expression including collagen type II, aggrecan, and SOX9 [47]. The chondrogenic outcomes observed in the present study, including the expression of aggrecan and collagen type II confirmed by immunofluorescence, provide indirect evidence that biologically active TGF-β3 remained available to the cells throughout the 28-day culture period. Nevertheless, direct quantification of TGF-β3 release kinetics would further strengthen these findings, and TGF-β3 release profiling is planned as part of future investigations.

### 3.6. Cell Morphology and Cytocompatibility of the Scaffolds

**Figure 6:**
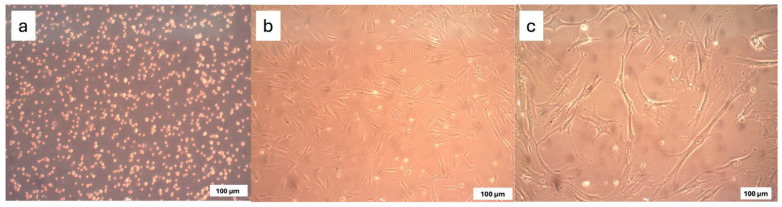
Representative light micrographs of chondrocytes showing (**a**) cells in suspension culture and (**b**,**c**) cells adhered and spread on a tissue culture plate (2D culture) (scale bar = 100 µm).

The morphology of chondrocytes varied depending on the culture conditions (Figure 6). Freshly prepared chondrocytes maintained in suspension exhibited a spherical morphology consistent with the native chondrocyte phenotype (Figure 6a). Following monolayer expansion on standard tissue culture plates, cells adopted an elongated, fibroblast-like morphology characteristic of chondrocyte dedifferentiation in 2D culture (Figure 6b,c) [48]. This morphological shift underscores the importance of three-dimensional scaffold systems that preserve the native rounded morphology for cartilage tissue engineering applications.

**Figure 7:**
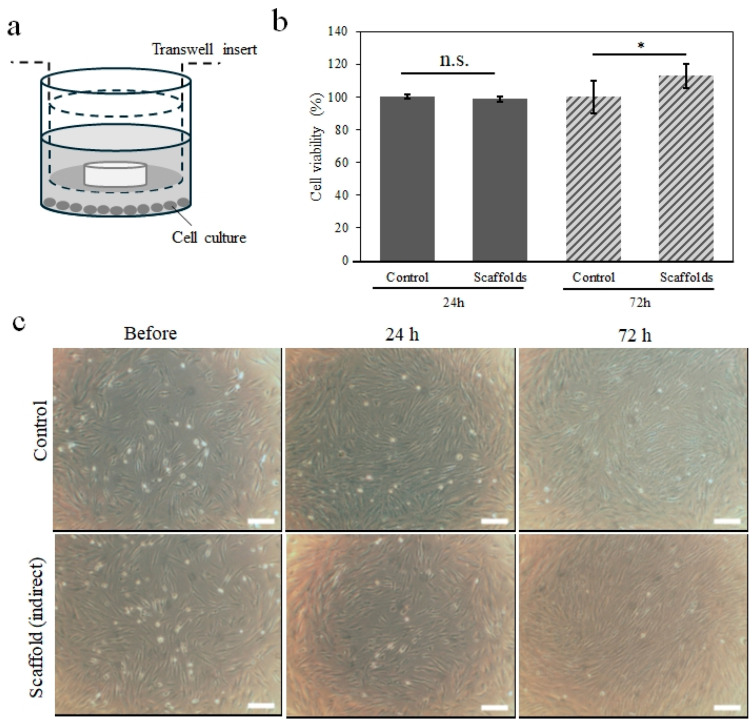
In vitro cell viability assessment. (**a**) Schematic illustration of the indirect contact culture system using a transwell insert, allowing soluble factors released from TGF-β3-loaded scaffolds to interact with cells without direct contact. (**b**) Cell viability after 24 and 72 h of indirect exposure to TGF-β3-loaded scaffolds evaluated by the MTT assay, expressed as a percentage relative to the scaffold-free control at the corresponding time point (defined as 100%). Data are presented as mean ± SD (*n* = 3). No significant difference in cell viability was observed between the control and scaffold groups at 24 h (unpaired two-tailed Student’s *t*-test, *p* > 0.05; n.s.), whereas a significant increase in cell viability was detected at 72 h (* *p* = 0.026; *p* < 0.05). (**c**) Representative phase-contrast micrographs of cells before treatment and after 24 and 72 h of indirect culture. Scale bars = 100 μm.

The cytocompatibility of the TGF-β3-loaded CS/SF/AV/Mimosa composite hydrogel scaffolds was evaluated using an in vitro indirect contact cytotoxicity test with a transwell system (Figure 7a). MTT assay results showed no significant difference in cell viability between the scaffold-treated and scaffold-free control groups at 24 h (98.8 ± 1.5% vs. 100.0 ± 1.2%, *p* > 0.05), indicating the absence of early cytotoxic effects (Figure 7b). At 72 h, cell viability in the scaffold-treated group (112.9 ± 7.3%) was significantly higher than that of the control (100.0 ± 9.8%, *p* = 0.026), reflecting a time-dependent increase in cellular metabolic activity. Representative phase-contrast micrographs confirmed preserved cell morphology at both time points (Figure 7c). The elevated cell viability at 72 h is likely attributable to soluble factors released from the scaffold. In particular, TGF-β3 and bioactive compounds from the *Aloe vera* and Mimosa components may have been gradually released into the surrounding medium, stimulating chondrocyte proliferation and activity even without direct cell–scaffold contact [49]. Collectively, these findings confirm that the composite hydrogel scaffold is cytocompatible and supports chondrocyte viability over time. These results highlight its potential for cartilage tissue engineering applications.

### 3.7. Morphological Evaluation of Chondrocytes Cultured on Three-Dimensional Scaffolds

**Figure 8:**
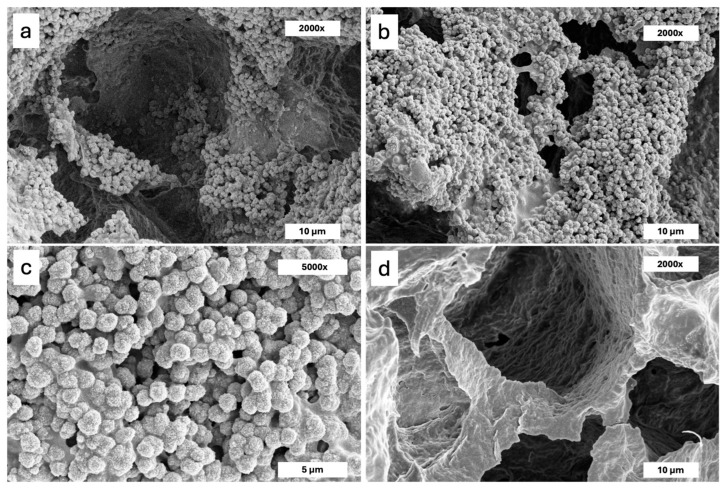
FESEM images of cell-seeded scaffolds after 28 days of culture (**a**–**c**) and a cell-free scaffold cultured under the same conditions (**d**). In cell-seeded scaffolds, rounded cell clusters with extensive extracellular matrix deposition were observed covering pore walls and bridging interconnected pores at 2000× (**a**,**b**) and 5000× (**c**) magnification, consistent with chondrocyte morphology in a three-dimensional environment. The cell-free scaffold (**d**) retained its original porous architecture without evidence of cell attachment or ECM deposition at 2000× magnification. Scale bars: (**a**,**b**,**d**) 10 µm; (**c**) 5 µm.

The surface morphology and cell–scaffold interactions after 28 days of culture were examined by FESEM (Figure 8). The cell-seeded scaffolds exhibited extensive coverage of pore walls by cellular structures and ECM, indicating successful cell adhesion and colonization within the three-dimensional scaffold architecture (Figure 8a–c). At 2000× magnification (Figure 8a), scaffold pores were partially filled with dense biological material, suggesting progressive cell infiltration and matrix accumulation within the interconnected porous network. At the same magnification (Figure 8b), cells and deposited ECM bridged adjacent pores, forming continuous networks that reflect effective cell migration and spatial integration throughout the scaffold. At higher magnification (5000×, Figure 8c), the surface was characterized by densely packed, rounded granular aggregates consistent with chondrocytes surrounded by pericellular ECM, indicative of chondrogenic phenotype maintenance. Similar ultrastructural features have been reported for chondrocytes cultured within hydrogel and porous polymeric scaffolds and are associated with proteoglycan-rich pericellular matrix accumulation during cartilage-like tissue formation [50]. In contrast, the cell-free scaffold retained its original porous architecture without evidence of ECM deposition or cellular coverage (Figure 8d, 2000×), confirming that the morphological changes observed in Figure 8a–c were cell-mediated rather than artifacts of scaffold degradation.

The rounded, clustered cell morphology observed under three-dimensional culture conditions contrasts markedly with the flattened, fibroblast-like morphology typically associated with chondrocyte dedifferentiation in two-dimensional monolayer cultures [51]. Maintenance of a rounded morphology is widely recognized as a hallmark of chondrocytes in three-dimensional microenvironments and is closely associated with preservation of the chondrogenic phenotype [52]. The clustered arrangement observed in this study further suggests that the scaffold provided appropriate mechanical confinement and cell–matrix interactions, supporting spatial organization and phenotype maintenance.

Collectively, the FESEM findings demonstrate that the scaffold supports chondrocyte adhesion, rounded morphology, spatial organization, and ECM deposition—features that closely resemble the native cartilage microstructure in which chondrocytes reside within a dense extracellular matrix with limited spreading. These results are consistent with current cartilage tissue engineering strategies, emphasizing the importance of three-dimensional scaffold architecture in maintaining chondrocyte phenotype and promoting cartilage-like tissue formation [53,54].

### 3.8. Immunofluorescence Staining of Collagen Type II and Aggrecan

**Figure 9:**
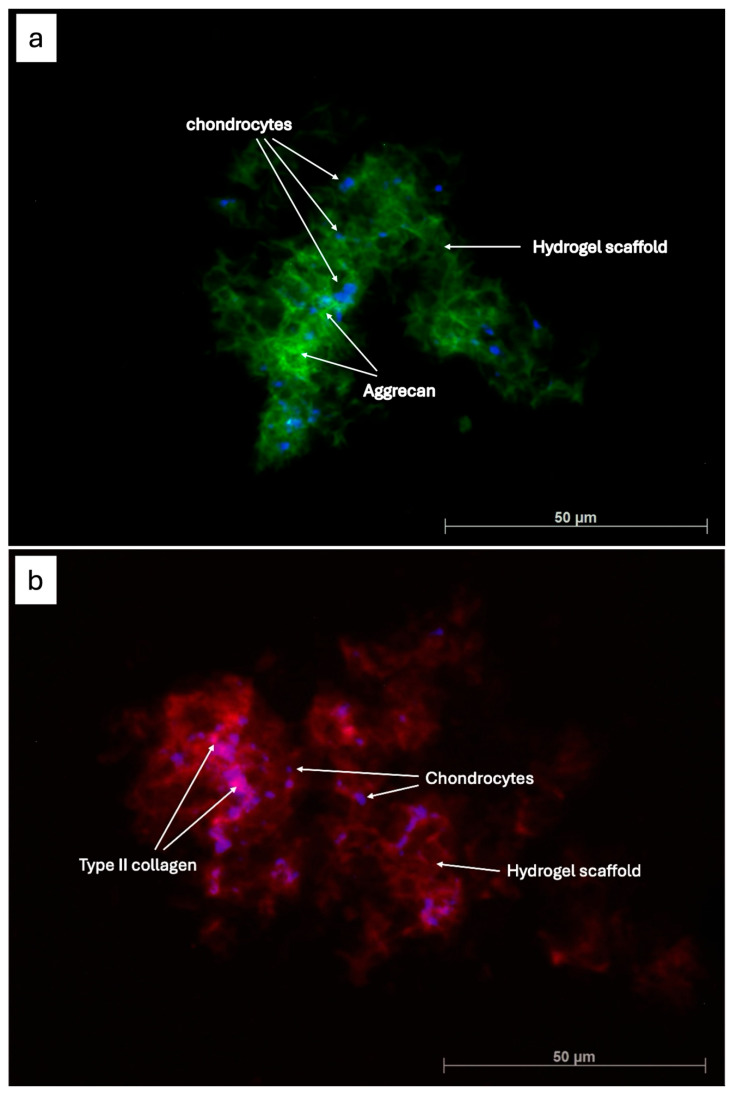
Immunofluorescence images of chondrocytes cultured on the hydrogel scaffold at 28 days. (**a**) Aggrecan expression (green, Alexa Fluor 488) surrounding chondrocyte clusters within the scaffold matrix, indicating proteoglycan-rich extracellular matrix deposition. (**b**) Collagen type II expression (red, Alexa Fluor 647) localized around chondrocytes and distributed throughout the scaffold matrix, confirming the presence of cartilage-specific collagen. Nuclei were counterstained with DAPI (blue). Scale bar = 50 μm.

Immunofluorescence staining was performed to evaluate cartilage-specific extracellular matrix production by chondrocytes cultured on hydrogel scaffolds at 28 days (Figure 9). Aggrecan (green, Alexa Fluor 488) was clearly detected surrounding chondrocyte clusters within the scaffold matrix, indicating substantial proteoglycan-rich matrix accumulation consistent with pericellular and territorial matrix deposition (Figure 9a). Collagen type II (red, Alexa Fluor 647) was similarly localized around chondrocytes and distributed throughout the scaffold matrix, confirming cartilage-specific collagen deposition within the three-dimensional scaffold environment (Figure 9b). DAPI-stained nuclei (blue) revealed that chondrocytes formed clustered aggregates within the scaffold, reflecting active cell proliferation and spatial organization within the porous structure.

The co-localization of aggrecan and collagen type II with chondrocyte clusters confirms active production of the two principal components of hyaline cartilage matrix and indicates maintenance of the chondrogenic phenotype. These results are consistent with previous reports showing that three-dimensional hydrogel-based systems support aggrecan and collagen type II expression more effectively than two-dimensional monolayer cultures, where chondrocytes typically undergo dedifferentiation [55]. The distribution of these matrix markers around rounded cell clusters further suggests that the scaffold microenvironment supports phenotype maintenance through cell–matrix interactions, in which a rounded cell morphology regulates cytoskeletal dynamics and downstream chondrogenic gene expression, including SOX9, ACAN, and COL2A1 [51,56]. The pronounced matrix deposition observed at 28 days is likely further supported by sustained TGF-β3 release from the scaffold. TGF-β3 is a well-established chondrogenic factor that promotes SOX9-dependent signaling, thereby enhancing proteoglycan and collagen type II synthesis within three-dimensional scaffold environments [57]. Together, these findings support the potential of this scaffold system to promote stable cartilage-like tissue formation in vitro through the combined effects of a three-dimensional architecture and controlled chondrogenic factor delivery [58].

## 4. Conclusions

In this study, a multifunctional composite hydrogel scaffold composed of chitosan, silk fibroin, *Aloe vera* extract, and a Mimosa complex was successfully developed as a bioactive platform for cartilage tissue engineering. The freeze-dried scaffolds exhibited a porous and interconnected three-dimensional architecture suitable for cell infiltration and matrix deposition. FTIR and XRD analyses confirmed successful incorporation of all components into the final formulation, with the Mimosa complex displaying a predominantly amorphous pattern consistent with effective inclusion complex formation within the HPβCD cavity.

Cytocompatibility evaluation confirmed that TGF-β3-loaded scaffolds were non-cytotoxic and supported sustained chondrocyte metabolic activity. The three-dimensional environment preserved the characteristic rounded chondrocyte morphology, while FESEM analysis demonstrated extensive cell adhesion, infiltration, and extracellular matrix deposition within the scaffold pores. Immunofluorescence staining further revealed time-dependent upregulation of aggrecan and collagen type II, confirming progressive cartilage-specific matrix formation.

Overall, this composite hydrogel scaffold integrating a biomimetic polymeric matrix with localized TGF-β3 delivery represents a promising platform for cartilage regeneration. Future in vivo studies using appropriate cartilage defect models are warranted to validate its regenerative potential prior to clinical translation.

## Data Availability

The original contributions presented in this study are included in the article. Further inquiries can be directed to the corresponding authors.
